# Innovative Strategies to Facilitate Patient-Centered Research in Multiple Chronic Conditions

**DOI:** 10.3390/jcm10102112

**Published:** 2021-05-14

**Authors:** Tullika Garg, Courtney A. Polenick, Nancy Schoenborn, Jane Jih, Alexandra Hajduk, Melissa Y. Wei, Jaime Hughes

**Affiliations:** 1 Department of Urology, Department of Population Health Sciences, Geisinger, Danville, PA 17822, USA; 2 Geriatric Psychiatry Program, Department of Psychiatry, University of Michigan, Ann Arbor, MI 48109, USA; cpolenic@med.umich.edu; 3 Division of Geriatric Medicine and Gerontology, Johns Hopkins University School of Medicine, Baltimore, MD 21205, USA; nancyschoenborn@jhmi.edu; 4 Division of General Internal Medicine and Multiethnic Health Equity Research Center, University of California San Francisco, San Francisco, CA 94143, USA; jane.jih@ucsf.edu; 5 Section of Geriatrics, Department of Internal Medicine, Yale School of Medicine, New Haven, CT 06510, USA; Alexandra.hajduk@yale.edu; 6 Division of General Internal Medicine and Health Services Research, David Geffen School of Medicine, University of California, Los Angeles, CA 90095, USA; melissa.y.wei@gmail.com; 7 Center for the Study of Healthcare Innovation, Implementation and Policy, VA Greater Los Angeles Healthcare System, Los Angeles, CA 90073, USA; 8 Department of Population Health Sciences, Duke University School of Medicine, Durham, NC 27705, USA; jaime.hughes@duke.edu

**Keywords:** multimorbidity, chronic disease, patient-centered care, aging

## Abstract

Multiple chronic conditions (MCC) are one of today’s most pressing healthcare concerns, affecting 25% of all Americans and 75% of older Americans. Clinical care for individuals with MCC is often complex, condition-centric, and poorly coordinated across multiple specialties and healthcare services. There is an urgent need for innovative patient-centered research and intervention development to address the unique needs of the growing population of individuals with MCC. In this commentary, we describe innovative methods and strategies to conduct patient-centered MCC research guided by the goals and objectives in the Department of Health and Human Services MCC Strategic Framework. We describe methods to (1) increase the external validity of trials for individuals with MCC; (2) study MCC epidemiology; (3) engage clinicians, communities, and patients into MCC research; and (4) address health equity to eliminate disparities.

## 1. Introduction

Multiple chronic conditions (MCC), defined by the U.S. Department of Health and Human Services (HHS) as “the presence of two or more co-existing chronic conditions that last one year or longer and require ongoing medical attention and/or limit activities of daily living”, represent one of today’s most pressing healthcare concerns. Nearly 25% of all Americans and 75% of older Americans over 65 years have MCC [1]. MCC are associated with poor health-related quality of life, increased healthcare use and costs, and care fragmentation [2]. As clinical care for individuals with MCC is often complex, condition-centric, and poorly coordinated across multiple specialties and healthcare settings, there is an urgent need for multidisciplinary, patient-centered research to redesign care for this burgeoning population.

In response to the growing need to advance MCC science, the National Institute on Aging (NIA) funded a nationwide MCC research network of academic medical centers and integrated health systems. The Advancing Geriatrics Infrastructure and Network Growth (AGING) Initiative was formed in 2014 as an infrastructure network to further team science in MCC research [3]. In 2018, the AGING Initiative launched the MCC Scholars program with the goal of growing and sustaining a community of multidisciplinary investigators committed to MCC research.

This manuscript, written in collaboration by the inaugural cohort of MCC Scholars, describes innovative, patient-centered approaches to MCC research. We organized our discussion around four research objectives described in the HHS MCC Strategic Framework: (1) increasing the external validity of trials; (2) MCC epidemiology; (3) increasing clinical, community, and patient-centered health research; and (4) addressing disparities in MCC populations [4].

## 2. Increasing the External Validity of Trials

Individuals with MCC are often excluded from clinical trials due to medical complexity. The implications of such omissions are concerning as clinical guidelines and evidence-based programs continue to focus on a single disease rather than addressing a patient-centered holistic picture of interconnected chronic conditions. Ideally, clinical trials and other research studies should be inclusive of medically complex individuals across the lifespan; however, when enrollment is lacking, other innovative approaches such as patient-centered outcomes research, pragmatic trials, and engagement principles from implementation science may help to fill evidence gaps.

### 2.1. Patient-Centered Outcomes Research

The outcomes measured in clinical trials are often not the outcomes that patients value most. For example, almost all existing prognostic indices focus on mortality as the outcome [5]. However, a study of older adults showed that 76% ranked functional independence, rather than survival, as the most important health outcome [6]. Another study found that patients with MCC who manage hypertension and diabetes were interested not only in traditional outcomes such as preventing long-term cardiovascular complications, but also in emotional health and well-being, care coordination, and health care navigation [7]. Focusing on patient-centered outcomes is particularly important in MCC given the complex interactions between conditions and their respective therapies.

In addition to engaging patients with MCC to define research outcomes a priori, it is important to assess how individuals prioritize outcomes within the context of their MCC. Individuals with MCC may have different values and preferences regarding specific outcomes since they are more likely to encounter treatment burden and side effects [8]. A number of studies have examined preferences of patients with MCC regarding the benefits and harms of specific treatments using stated-preference research methods. These innovative methods allow for quantification of patients’ preferences for various outcomes, which can then be combined with the estimated likelihood of each outcome to produce individualized benefit/harm assessments [9,10].

### 2.2. Pragmatic Trials

Unlike traditional clinical trials that occur within tightly controlled settings, pragmatic trials collect real-world data in a variety of settings and integrate multiple stakeholders (e.g., decision-makers and end users) throughout the process. Pragmatic trials are well-suited to improving the external validity of research findings for patients with MCC, due to less restrictive inclusion and exclusion criteria. Pragmatic trials allow for the evaluation of complex interventions in settings that more closely resemble the daily realities of managing MCC. For example, a recent pragmatic trial examined a physical activity intervention in over 2700 patients with chronic obstructive pulmonary disease, a burdensome condition associated with medical complexity and poor quality of life [11]. The study evaluated both traditional and patient-centered outcomes, and had few exclusion criteria, allowing for a cohort that reflected real-world patients and experiences. Researchers may access resources provided by the NIH Collaboratory and in tools such as the Readiness Assessment for Pragmatic Trials (RAPT) model to assess readiness for a pragmatic trial.

### 2.3. Implementation Science

Implementation science is defined as “the study of methods to promote the adoption and integration of evidence-based practices, interventions, and policies into routine health care and public health settings to improve the impact on population health” [12]. A central focus of implementation science is identifying the conditions under which translation of existing evidence is most feasible and effective. Implementation researchers select, refine, and test implementation strategies, which are tools and methods used to implement evidence into practice [13,14].

Three key implementation science principles are especially relevant to MCC research. First, engaging multilevel stakeholders, particularly the end users of programs—patients, caregivers, and providers—can help inform adaptations in order to maximize the fit between a program, its users, and setting. Second, the concept of designing for dissemination encourages researchers to begin at the end by considering long-term dissemination and implementation when designing and testing clinical interventions. These two principles merge into the concept of human-centered design, which aims to evaluate the usability of an intervention as well as its implementation strategies [15].

Understanding and addressing barriers and facilitators of implementation may increase the availability and accessibility of effective interventions to individuals with MCC.

## 3. Approaches to MCC Epidemiology

Developing methods to accurately assess MCC prevalence and incidence trends, particularly in underserved populations, is foundational to designing patient-centered care and measuring impacts. Current methods for MCC measurement are fluid due to a lack of criteria for the conditions that should be included in definitions of MCC. As there is no set methodology, we highlight a variety of approaches including patient-reported measures, patient-driven health information exchanges, claims-based tools, electronic health records, and ecological momentary assessment. Method selection must be congruent with the purpose of the project and the relevant stakeholders.

### 3.1. Patient-Reported MCC Measurement

Traditional measures of MCC include simple (i.e., unweighted) disease count and comorbidity indices designed for risk-adjustment in hospitalized patients. Comorbidity indices were originally developed to predict mortality, healthcare cost, and use, yet they are frequently applied to patient-centered outcomes such as health-related quality of life (HRQOL) despite not being designed for this purpose.

Patient-centered measures such as HRQOL are ideal for measuring the impacts of MCC as they are holistic and capture the cumulative impact of disease burden. Among the most comprehensive and validated MCC measures using a patient-reported approach is the multimorbidity-weighted index (MWI) [16]. MWI includes impactful prevalent and rare chronic conditions that are weighted by their impact on physical HRQOL, a universally valued patient-centered outcome. Because conditions are weighted to physical functioning in community-dwelling adults, MWI represents both the cumulative burden of disease and physical functioning. Other advantages include having the broadest distribution at both the low and high ends of MCC compared with traditional metrics, and extensive prospective and external validation for downstream consequences in several different cohorts including in Medicare beneficiaries and the nationally-representative Health and Retirement Study [17,18,19,20].

In addition, treatment burden is increasingly recognized as an important patient-centered measure in medically complex patients. Treatment burden is defined as “the work of being a patient and how it impacts patients’ functioning and well-being” [21]. The concept of treatment burden incorporates a constant tension between the amount of healthcare work in which patients engage and the resources available to accomplish the work. Healthcare work may include activities such as coordinating clinic appointments, organizing transportation, managing medications, among others. Older adults with MCC spend five to eight hours per day engaged in health-related activities [22]. Currently, multiple patient-reported measures are available to gauge treatment burden including the Treatment Burden Questionnaire [23], Multimorbidity Treatment Burden Questionnaire [24], and the Patient Experience with Treatment and Self-Management (PETS) measure [25].

### 3.2. Patient-Driven Health Information Exchanges

Patient-driven health information exchanges (HIEs) enable streamlined data collection in near real-time from multiple sources, including the electronic health record, pharmacy data, patient-reported data, and patient-generated data from smart medical devices and wearables. HIEs, such as Hugo (hugo.health) and CareEvolution (careevolution.com), present an exciting opportunity for MCC researchers to access diverse types of data from patients, providers, and health systems. In accordance with the 21st Century Cures Act, HHS and the Centers for Medicare and Medicaid Services issued regulations requiring public and private entities to share health information between patients and other parties while maintaining data privacy and security. These regulations are expected to expand HIEs in the coming years.

There are many benefits of using HIEs to conduct MCC research. HIEs collate longitudinal medical record data from multiple sources into one central repository. HIE patient-users, not health systems, own their health data and may view and share it as they wish, including with researchers. HIE applications have screening capabilities for eligibility criteria and allow researchers to contact eligible users with invitations to participate in research, including individuals residing in underserved areas. HIEs enable near real-time collection of patient-reported data, including for patient-centered outcomes relevant to MCC researchers such as physical function. The app-based interface eases the burden of serial longitudinal outcome assessment and may decrease loss to follow-up. Syncing health monitoring devices and wearables may decrease research-related burdens for participants and research personnel. All data collected in the application are viewable to patient-users, which may increase engagement in health care and MCC research activities.

Despite the promise that patient-driven HIEs may hold for MCC research, the technology is nascent and there are new concerns regarding the additional work of managing HIE data and the emotional toll of ongoing access to one’s health data [26]. In addition, despite the fact that three-quarters of older adults in the United States use the Internet, and the majority of older adults now own a smartphone, a digital divide persists for older adults regarding comfort with and acceptability of health information technology [27,28]. The divide is particularly marked among those of very advanced age or low educational attainment [29]. Appropriate testing of feasibility and acceptability of HIE, as well as feedback from research participants with MCC, is still needed.

### 3.3. Measuring MCC Using Claims Data

Several publicly available electronic tools may be helpful to researchers describing MCC epidemiology using claims data. The Agency for Healthcare Research and Quality Clinical Classifications Software categorizes ICD-9 and ICD-10 diagnosis codes into defined conditions. The Chronic Condition Indicator tool is organized by body system, and typically used in conjunction with the Clinical Classifications Software to form discrete chronic conditions. Both tools are available for ICD-9 codes but the ICD-10 codes remain in beta testing. We successfully used these tools with claims and electronic health record data [30].

For Medicare and Medicaid data, the Chronic Conditions Data Warehouse has predefined algorithms to identify 27 common chronic conditions and 40 other chronic or potentially disabling conditions. The ICD-9 and ICD-10 diagnosis codes are freely available for download, as are lists of references that inform each algorithm. These chronic condition measurements count individual conditions, but do not incorporate disease severity or impact on functional status.

The previously discussed MWI was also mapped to ICD-9 codes and is validated for use in administrative data [31]. The index weights individual conditions in order to capture the relationships between accumulation of chronic conditions and physical functioning that are not captured by simple condition counts.

Certain chronic conditions, though clearly associated with patient suffering and healthcare use, may be unrecognized as important chronic conditions and may also be underreported in claims data. For example, conditions such as chronic pelvic pain, chronic prostatitis, urinary incontinence, irritable bowel syndrome, and others may be poorly captured by diagnosis codes and/or procedure codes in claims data. Further research is needed to characterize these types of chronic conditions and to better identify them in claims data in order to measure impact.

### 3.4. Measuring MCC from Electronic Health Record Data

Electronic health records (EHRs) have proliferated across healthcare settings over the last decade. EHR data offer several advantages but also challenges compared with claims data and observational studies. The advantages include providing details of clinical encounters including unstructured data from clinic notes and large volumes of structured real-time flowsheet data (e.g., lab values, vital signs, and telemetry). Despite the depth of data available, the breadth may be limited due to domains that are inconsistently assessed or missing, making it difficult to examine demographics, socioeconomic status, and physical function. Nonetheless, EHRs confer opportunities for linkage across health systems, which would facilitate MCC research across diverse populations and geographies. For example, the Health Care Systems Research Network (HCSRN) is a national network of twenty U.S. health systems covering over 28 million unique patients [32]. The HCSRN member sites share a standardized data model called the Virtual Data Warehouse (VDW). As EHR data structure varies across different providers and systems, data models such as the VDW allow researchers to write extraction code that can be executed at each member site to yield a standardized dataset.

### 3.5. Ecological Momentary Assessment

Successfully managing chronic conditions involves adherence to self-management regimens and healthy behaviors, including diet, sleep, and physical activity. These activities occur in social, environmental, and medical contexts, and understanding an individual’s context may enhance adherence. Ecological momentary assessment (EMA) is used to collect rich context-specific data. EMA is characterized by real-time, repeated measures (e.g., activity, pain, mood, and symptoms) and can be delivered via telephone, smartphone, or tablet.

The ecological nature of EMA methods offers several advantages for data collection. First, these methods reduce the recall bias associated with retrospective questionnaires. Second, repeated assessments enhance the understanding of intra-individual daily symptom and behavior variability over time. Third, researchers are able to measure associations between an individual’s context and behaviors, symptoms, and outcomes because data are collected in a patient’s natural environment. These advantages may be particularly relevant to MCC populations whose symptoms can vary across time and context. Finally, EMA can inform ecological momentary interventions that are tailored to a patient’s needs and preferences, and can be delivered based on time, location, or symptom onset [33].

## 4. Increasing Clinical, Community, and Patient-Centered Health Research

Both the Institute of Medicine and the Patient-Centered Outcomes Research Institute have emphasized the importance of engaging stakeholders such as patients, communities, and organizations that have a direct interest in the processes and/or outcomes of research. Incorporating stakeholders in MCC research augments efforts to design and implement programs that improve the lived experiences and health outcomes of individuals with MCC. Below, we highlight how to engage patient and caregiver perspectives into the design and conduct of MCC research.

### 4.1. Engaging MCC Patients in Research Design

Best practices for stakeholder engagement can be leveraged to include MCC patients in setting research priorities and selecting research outcomes. For example, a patient and a caregiver stakeholder and an investigator group used a six-step process to establish MCC research priorities [7]. The research team iteratively narrowed down research questions and then restated them in patient-friendly language. Then, through two sets of focus groups, MCC patients and caregivers selected the top two research questions and the related outcomes of interest.

The use of crowdsourcing on social media platforms (i.e., directly soliciting user input) may also help to improve the management of MCC for both patient and caregiver stakeholders. For example, research analyzing health communications within diabetes-related Facebook pages found that crowdsourcing appears to encourage commenting among patients, which may enhance opportunities for peer-based emotional and informational support [34]. Similarly, an intervention in which caregivers of individuals with Alzheimer’s disease joined peer support social networks through “friendsourcing” was linked to higher perceptions of emotional and informational support, along with reduced levels of caregiver burden and perceived stress [35].

Despite these ongoing efforts to engage patients with MCC into research design, much of the existing evidence remains disease-specific and may not be generalizable to individuals with MCC. One potential solution is for providers to incorporate patient feedback while applying existing evidence. In the Patient Priorities Care model, patients with MCC identify specific health priorities, and clinicians seek to align medical decision-making to achieve the patient-identified health priorities. Patient Priorities Care is feasible and associated with reduced treatment burden and unwanted health care when compared to usual care [36].

### 4.2. Engaging Dyads and Caregivers in MCC Research

A key group of stakeholders is the family members and friends who provide unpaid care to individuals with MCC. Informal caregivers are essential in the long-term management of MCC; yet, they face numerous challenges such as insufficient knowledge about the patient’s conditions, uncertainty and worry about medication management and side effects, and poor communication and coordination across health care teams [37]. Informal caregivers are also often responsible for carrying out medical and nursing tasks (e.g., injections and wound care), though they often lack formal training in these tasks and find them to be difficult and stressful [37].

To design effective, personalized interventions for patients with MCC, it is imperative to engage their caregivers in research. Unfortunately, few studies have considered caregiver experiences and outcomes in the context of MCC [37]. Caregivers may differ in their needs and resources, daily routines and stressors, quantity and quality of social support, and emotions toward the care role, all of which vary widely with the characteristics of the caregiver (e.g., gender and relationship to the patient) and patient (e.g., age and total number and combinations of chronic conditions). It is also critical to consider factors associated with risk and resilience among caregivers of individuals with MCC. Prospective studies that identify factors that mitigate or intensify adverse outcomes will help to determine intervention targets and allocate limited resources [37].

MCC research that focuses on the care dyad as the unit of analysis, rather than individual caregivers and patients, captures both patient and caregiver perspectives as well as their interpersonal dynamics. Caregiving relationships can be either supportive or conflictual, which may have downstream implications for dyadic outcomes [38]. Furthermore, the presence of MCC among both patients and caregivers may shape health trajectories. For example, in a large study of older couples in the United States, individual and couple patterns of MCC were associated with important outcomes over time including depressive symptoms and functional disability [39,40]. Considering these interpersonal processes allows for a more nuanced picture of MCC management, along with the unique strengths and vulnerabilities within the dyad.

## 5. Addressing Disparities in MCC Populations

Inadequate representation of individuals from diverse backgrounds or underserved populations in research runs in opposition to the principle of justice as outlined in the Belmont Report. Relevant to MCC research, health disparities by social determinants of health such as age, gender identity, race/ethnicity, geography, and unmet health-related social needs are important considerations among individuals with MCC. For example, rurality and social deprivation are associated with high rates of MCC [41].

To address the complexity of MCC in health disparity populations, multi-level research is needed to define the environmental, sociocultural, behavioral, and biological factors that contribute to MCC in diverse settings. These data are foundational to designing and implementing scalable and culturally responsive interventions for clinical and community contexts. Moreover, it is imperative that MCC research is equitable and inclusive of NIH priority populations across the lifespan.

Diverse stakeholder perspectives are critical to effective MCC disparities research. Engaging with diverse stakeholders requires a respectful approach that aligns with the culture, norms, and values of the group. Starting with a respectful approach with active listening helps to build trust between participants and research teams, and their wider community. Researchers must consider the added burdens of research activities within the larger context of MCC and health-related tasks. As researchers learn more about facilitators and barriers to research participation, flexible research workflows can be iteratively adjusted and personalized to individual circumstances. Patient-centered consents and research materials that are culturally responsive, affirming, and in multiple languages reduce barriers to participation including for those who speak languages other than English. When developing research materials and training research staff, researchers should consider the education level and health literacy of the research population to ensure meaningful, informed consent and participation. Multiple touch points and modalities for research participation—by phone, paper, in-person, electronically, or others—reduce the burdens of research and augment patient autonomy as participants determine how and when they enroll in research.

## 6. Discussion

MCC are a growing challenge in healthcare due to the inherent complexity and the need for multidisciplinary and transdisciplinary approaches. There are few existing templates to address such challenges, and innovative patient-centered approaches deployed by diverse teams will be critical in advancing MCC research and clinical care.

**Figure 1:**
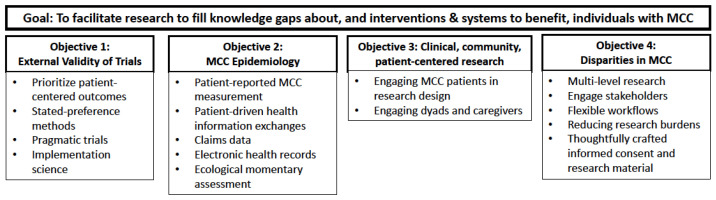
Innovative patient-centered methods for MCC research based on the HHS strategic framework. MCC: Multiple chronic conditions; HHS: U.S. Department of Health and Human Services.

We described specific methods corresponding to the research objectives of the HHS MCC Strategic Framework. The main research goal under the framework is to “facilitate research to fill knowledge gaps about, and interventions and systems to benefit, individuals with MCC”. Under this overarching goal, HHS lists four objectives: (1) address existing research gaps related to external validity of clinical trial data; (2) MCC epidemiology; (3) clinical, community, and patient-centered health research; and (4) MCC disparities. We highlighted patient-centered methods to address each of these four MCC research objectives, while remaining mindful of the limitations of existing methods for addressing the complexities of MCC (Figure 1).

In particular, understanding multilevel MCC impacts on patients, families, communities, and healthcare systems will be important for designing personalized care. No two patients with MCC are the same, with diverse conditions of varying severity, multifactorial social determinants, and differences in healthcare access and use preferences. Future innovations in MCC research methods and interventions will incorporate multilevel factors to understand the individualized context and related outcomes.

Team science will play an important role in advancing innovations for MCC research. MCC research is beyond multidisciplinary—it is transdisciplinary, as we create an altogether new field and approaches. Networks such as the AGING Initiative that gather diverse disciplines, researchers, stakeholders, and institutions will be instrumental in forming crosscutting collaborations that lead to creative methods [3]. Research networks addressing complex healthcare problems, including MCC, are recognizing the importance of patient and caregiver perspectives in formulating the most impactful research questions. For example, the AGING Initiative has a Patient/Caregiver Advisory Council that attends meetings, reviews grant proposals, identifies research priorities, and provides mentorship on stakeholder engagement. Finally, although not addressed at length in this manuscript, there is a significant need for education for trainees, researchers, and clinicians conducting MCC research and/or caring for patients with MCC. Given the innately multidisciplinary nature of caring for patients with MCC, helping clinicians to learn how to work effectively in interprofessional teams (e.g., subspecialists, pharmacists, physical therapists, and others) is an important priority.

Through the iterative processes of innovation, stakeholder engagement, and research mentorship, it is our hope that MCC research will continue to grow and evolve into a patient-centered transdisciplinary field that will serve as a model for other complex healthcare challenges.
